# Ratiometric Fluorescent Nanoprobe for Highly Sensitive Determination of Mercury Ions

**DOI:** 10.3390/molecules24122278

**Published:** 2019-06-19

**Authors:** Zhihui Luo, Hui Xu, Baogui Ning, ZeBin Guo, Na Li, Lina Chen, Guobao Huang, Charlie Li, Baodong Zheng

**Affiliations:** 1 College of Food Science, Fujian Agriculture and Forestry University, Fuzhou, Fujian 350002, China; lzhui_1980@163.com (Z.L.); xhuifst@163.com (H.X.); gzb8607@163.com (Z.G.); 2 Guangxi Key Laboratory of Agricultural Resources Chemistry and Biotechnology, College of Chemistry and Food Science, Yulin Normal University, Yulin, Guangxi 537000, China; ningbg1108@163.com (B.N.); ln19860622@126.com (N.L.); lnchen2018@163.com (L.C.); lzjx0915@163.com (G.H.); 3 Engineering Research Center of Marine Living Resources Integrated Processing and Safety Risk Assessment, Fuzhou, Fujian 350002, China; 4 Department of Environmental Toxicology, University of California-Davis, Davis, CA 95616, USA

**Keywords:** ratiometric fluorescent nanoprobe, dual-emission, mercury ions, determination

## Abstract

In this study, a novel dual-emission ratiometric fluorescent nanoprobe (RFN) was synthesized and ultilized for highly sensitive determination of mercury ions. In this nanoprobe, fluorescein isothiocyanate (FITC) doped silica (SiO_2_) served as a reference signal, FITC–SiO_2_ microspheres were synthesized and modified with amino groups, and then Au Nanoclusters (AuNCs) were combined with the amino groups on the surface of the FITC–SiO_2_ microspheres to obtain the RFN. The selectivity, stability, and pH of the RFN were then optimized, and the determination of mercury ions was performed under optimal conditions. The probe fluorescence intensity ratio (F_520_ nm/F_680_ nm) and Hg^2+^ concentration (1.0 × 10^−10^ mol/L to 1.0 × 10^−8^ mol/L) showed a good linear relationship, with a correlation coefficient of R^2^ = 0.98802 and a detection limit of 1.0 × 10^−10^ mol/L, respectively. The probe was used for the determination of trace mercury ion in water samples, and the recovery rate was 98.15~100.45%, suggesting a wide range of applications in monitoring pollutants, such as heavy metal ion and in the area of environmental protection.

## 1. Introduction

Mercury is commonly found in the environment and is one of the most toxic elements [1]. It cannot be degraded by microorganisms in water. Mercury and some of its salts are volatile, thus possessing high mobility, corrosivity, and carcinogenicity. It can cause cell division damage and permanent damage to the central nervous system through skin and respiratory system, and it is one of the most common environmental pollutants [2,3,4]. According to the United States Environmental Protection Agency, the maximum concentration of mercury ions in human daily drinking water should not exceed 10 nmol/L (ppb). Therefore, it is of great significance to establish a method with high sensitivity and selectivity for detecting mercury ions in the environment.

At present, the effective methods for determining mercury ions include atomic absorption spectroscopy, inductively coupled plasma mass spectrometry, inductively coupled plasma-atomic emission spectrometry, and so on [5,6,7]. However, to perform these with a high accuracy, these methods are time-consuming, expensive, and require professional operation. To overcome these shortcomings, the fluorescence (FL) method is often employed, as it has high sensitivity and selectivity, and is relatively easy to handle [8,9]. In recent years, the determination of mercury ions based on the establishment of fluorescence sensors has attracted much attention, including probes such as organic dyes [10,11], complexes [12], quantum dots [13], and peptides [14]. Compared with a single quencher, the dual-emission ratiometric fluorescent probe has the characteristics of large molar absorbance, good stability, strong anti-interference, and long emission wavelength, which proved to be an effective method for complex sample matrices, and it is the current direction for the research in mercury ion detection [15,16,17].

In this study, FITC–SiO_2_ was synthesized, and modified with amino groups, and then AuNCs was combined with the amino compounds on the surface of FITC–SiO_2_ microspheres to generate the RFN. By optimizing the interference, stability, and pH conditions of the RFN, The detection performance of the RFN on mercury ions was further explored and applied to the determination of water samples.

## 2. Results and Discussion

### 2.1. Fluorescence Spectra of RFN

**Figure 1:**
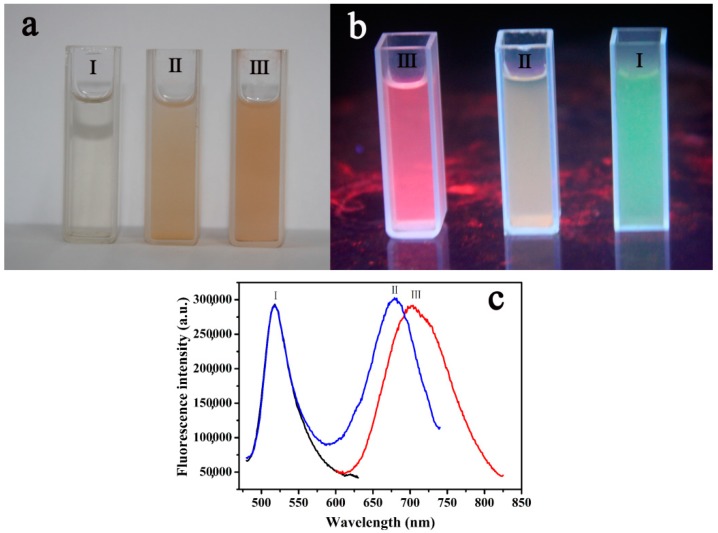
Photographs of the FITC–SiO_2_ (I), RFN (II), and AuNCs (III) in the sunlight (**a**) and under the ultraviolet lamp (**b**). The FL spectra of FITC–SiO_2_ (I), RFN (II), and AuNCs (III) (**c**).

Figure 1a shows the photographs of FITC–SiO_2_ (I), RFN (II) and AuNCs (III) under white light, and Figure 1b exhibits the corresponding photographs under ultraviolet light. As shown in these figures, the nanoparticles in various synthetic stages all exhibited excellent optical properties. The solutions of AuNCs and FITC–SiO_2_ showed red and green emissions, respectively, while the synthesized RFN (II) exhibited the complexed orange color. When the fluorescence spectra were subsequently measured by excitation wavelength of 380 nm, the maximum emission wavelengths of AuNCs and FITC–SiO_2_ were 698 nm and 520 nm, and the dual-emission peaks of the RFN were 520 nm and 680 nm, respectively. This indicated that the fluorescence wavelength of the AuNCs coupled to the FITC–SiO_2_ microspheres had undergone a blue shift, which might be due to the fact that the binding of amino compounds on the surface changed as a result of the activation effect of EDC.

### 2.2. Transmission Electron Microscopy (TEM) of RFN

**Figure 2:**
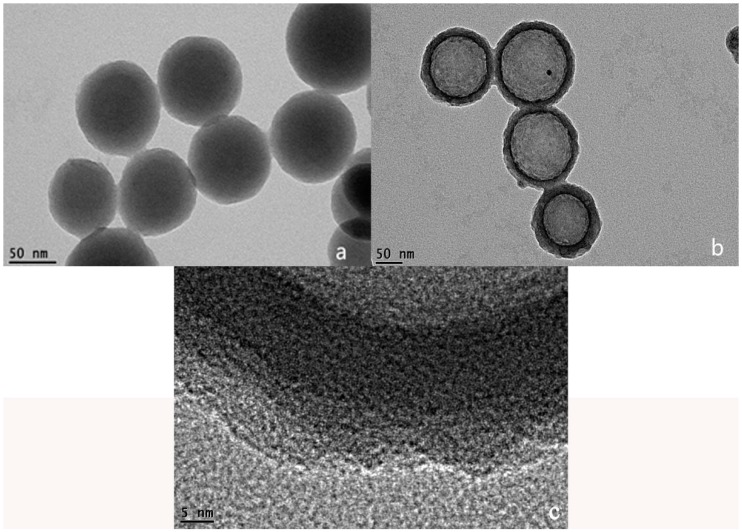
TEM of FITC–SiO_2_ (**a**), RFN (**b**), and high resolution transmission electron microscopy (HRTEM) image of the outer surface of the RFN (**c**).

To prove that AuNCs were successfully attached to the surface of FITC–SiO_2_ nanoparticles, the synthesized ratiometric fluorescent nanomaterials were characterized by TEM (Figure 2). Figure 2a shows the electron micrograph of SiO_2_–FITC, and Figure 2b shows the electron micrograph of RFN. As seen in the figures, the prepared FITC–SiO_2_ nanoparticles had a dimension of about 100 nm, while the particle size of RFN are 120 nm. It shows that the RFN had a very good dispersity. According to the literature [18,19], the particle size of the Au NCs was only about 1–2 nm, it was difficult to observe the AuNCs on the outer layer of FITC–SiO_2_ (Figure 2c) due to the aggregation of silica layer during the linking of AuNCs.

### 2.3. Energy Dispersive X-Ray Spectroscopy (EDS) of RFN

To further prove the AuNCs were successfully coupled with FITC–SiO_2_, the synthesized material was analyzed by electron scattering spectroscopy. Figure S1 shows the elements including oxygen, silicon, and gold on the RFN, confirming the AuNCs have been successfully link to the suface of FITC-SiO_2_.

### 2.4. Fluorescence Stability of RFN

**Figure 3:**
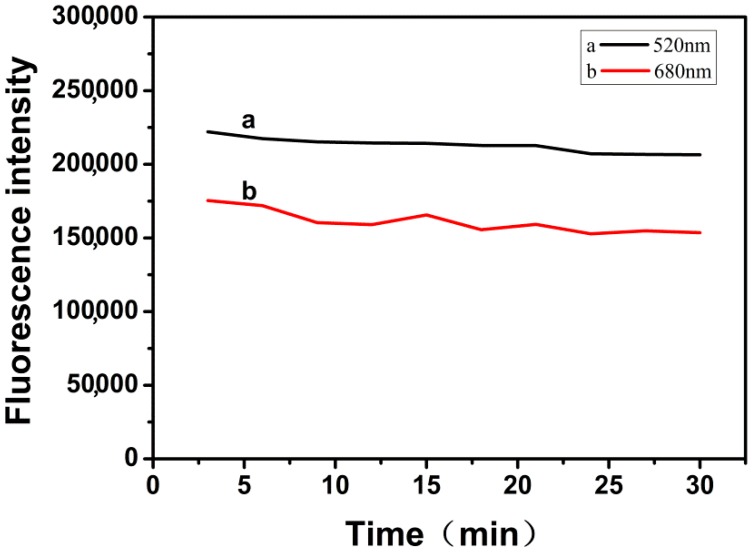
The fluorescence intensity of the RFN under 30 min.

To demonstrate the stability of the RFN, 150 μL of RFN and 500 μL of 2.5 × 10^−9^ moL/L Hg^2+^ were placed in a 4 mL cuvette, and 3.35 mL of water was added to adjust the volume. Under the same conditions, one group was measured every three minutes, and ten other groups were measured in parallel. As shown in Figure 3, the fluorescence intensity of the RFN varied only slightly at 520 or 680 nm, demonstrating the good stability of the RFN.

### 2.5. Effect of Buffer Solutions at Different pH Value on the RFN

**Figure 4:**
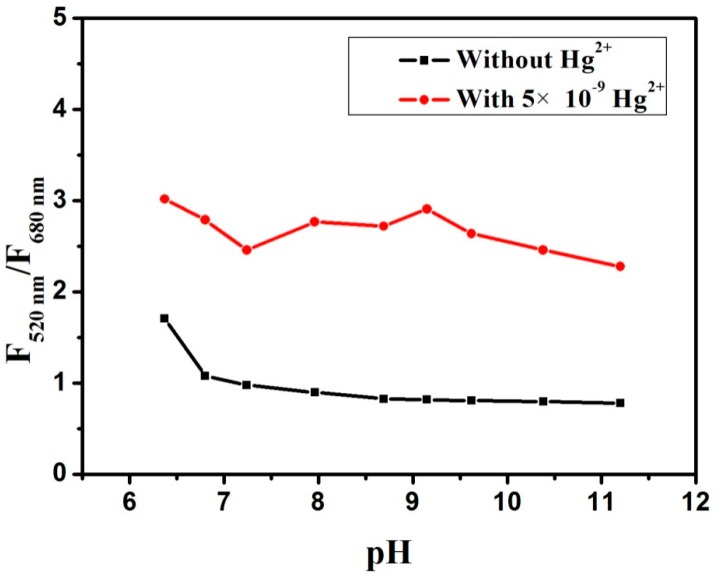
The F_520nm_/F_680nm_ intensity of RFN under the different of pH.

To study the effect of pH on the recognition of metal ions by RFN, we investigated the effect of buffer solutions at different pH values on the maximum fluorescence emission intensity of the RFN in the pH range of 6–11, with and without Hg^2+^. We selected the Britton-Robinson buffer system as the buffer solution and prepared various buffer solutions with different pH values in advance. The experiment was divided into two groups. For the first group, 100 μL of RFN was added directly to 3.4 mL of buffer solution at various pH values, and then 500 μL of 2.5 × 10^−9^ mol/L Hg^2+^ solution was added; for the other group, 100 μL of RFN was directly added to 3.4 mL of buffer solutions at various pH values without the addition of Hg^2+^ solution. The results are shown in Figure 4. The black square points represented the fluorescence intensity ratio of F_520nm_/F_680nm_ in the solutions of RFN with different pH values without the addition of Hg^2+^ solution, and the red dot represented the fluorescence intensity ratio of F_520nm_/F_680nm_ of the RFN after the addition of Hg^2+^ solutions. It can be seen from the figure that when Hg^2+^ was not added, the fluorescence intensity ratio decreased at pH = 6.37–7.24 and fluctuated slightly between pH = 7.24–10.38, having the highest intensity ratio and the best quenching effect at pH = 6.37. When Hg^2+^ was added, the fluorescence intensity ratio decreased between pH = 6.37–7.24 and pH = 9.15–10.38, increased with fluctuations between pH = 7.24–9.15, and the highest intensity was at pH = 9.15 with the best quenching effect.

### 2.6. Selectivity of the RFN to Hg^2+^

**Figure 5:**
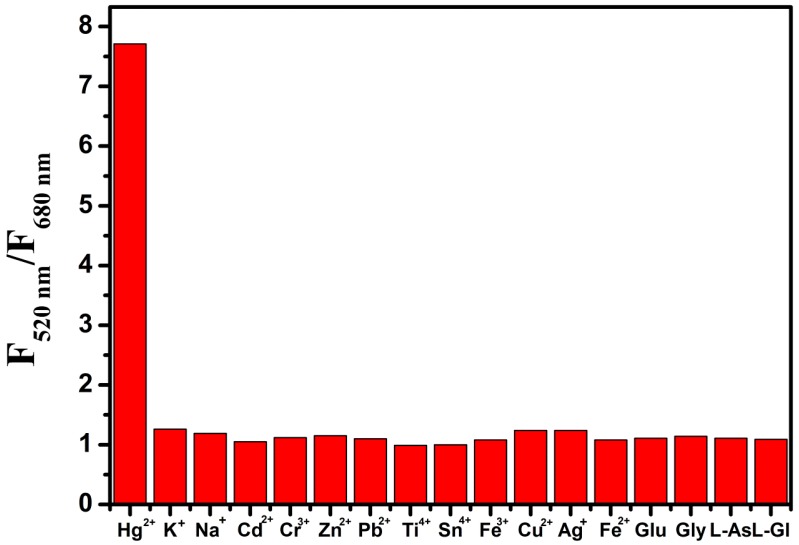
The F_520nm_/F_680nm_ intensity of RFN to various metal ions and amino acids (1.0 mM).

To demonstrate the selectivity of the RFN for the detection of mercury ions, 100 μL of the RFN was added to a 4 mL cuvette, followed by the addition of 500 μL of different types of common metal ions and amino acids, which were detected at room temperature for 5 min. As shown in Figure 5, among the interfering substance tested including K^+^, Na^+^, Cd^2+^,Cr^3+^, Zn^2+^,Pb^2+^,Ti^3+^, Sn^2+^,Fe^3+^, Cu^2+^, Ag^+^, Fe^2+^,Glu, Gly, L-As, L-Gl, only Hg^2+^ caused a significant decrease in fluorescence intensity, while other metal ions had almost no effect on the fluorescence spectra of the RFN. The dual-emission fluorescence ratio (F_520nm_/F_680nm_) was around 1, and only when Hg^2+^ was added, the F_520nm_/F_680nm_ reached 7.71. Under the same conditions, even if the concentration of other ions was much higher than that of Hg^2+^, the RFN could not be interfered, which could be used for selectively identify Hg^2+^ with low concentrations, resulting in quenching of the red fluorescence. That is, Hg^2+^ could quench the second peak of the fluorescence spectrum of the fluorescent probe, while other ions had no obvious quenching effect. This result indicated that the RFN has good selectivity and specificity.

### 2.7. Determination of Mercury Ions

**Figure 6:**
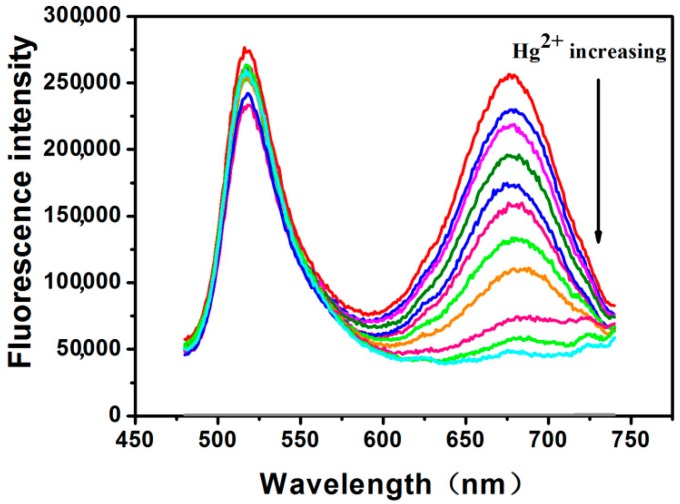
The FL spectra of RFN under different mercury concentrations (1 × 10^−10^, 2.5 × 10^−10^, 5 × 10^−10^, 7.5 × 10^−10^, 1 × 10^−9^, 2 × 10^−9^, 2.5 × 10^−9^, 3.5 × 10^−9^, 5 × 10^−9^,7.5 × 10^−9^, 1 × 10^−8^ mol/L) (λ_ex_ = 380 nm).

To test the feasibility of the RFN for detecting Hg^2+^, different concentrations of Hg^2+^ were added to the RFN and the change in fluorescence intensity was examined. Figure 6 is the change in fluorescence spectrum of 100 μL ratio fluorescent probe and 3.4 mL high purity water with the addition of 500 μL of variously concentrated Hg^2+^ solution. The double emission peaks of RFN were at 520 nm and 680 nm, respectively, and the maximum emission wavelength was 680 nm. With the increase of Hg^2+^ concentration, the fluorescence intensity of the second peak of RFN gradually weakened. The fluorescence intensity was completely quenched when the Hg^2+^ concentration reached 1 × 10^−8^ mol/L. The fluorescence intensity was highly sensitive at 680 nm Hg^2+^, and decreased with the increase of Hg^2+^ concentration. The mechanism of fluorescence quenching caused the metallophilic Hg^2+^–Au^+^ interactions in our experiment, because the surface of AuNCs with a small amount of Au^+^ should have strong and specific interactions with Hg^2+^, which leads to a serious quenching of the AuNCs fluorescence [19]. Moreover, there was no significant change in the peak at 520 nm throughout the experiment, indicating that the 520 nm emission peak could be used as an internal reference.

**Figure 7:**
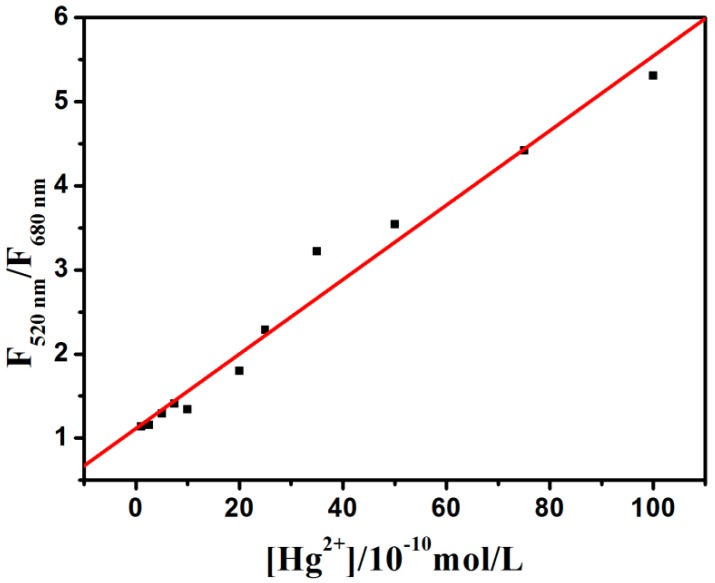
The F_520nm_/F_680nm_ intensity of RFN versus the concentration of Hg^2+^.

Figure 7 suggested that the Hg^2+^ exhibited a good linear relationship within the concentration range of 1.0 × 10^−10^ mol/L to 1.8 × 10^−8^ mol/L. The linear correlation coefficient was R^2^ = 0.98802, the linear regression equation was Y = 1.11368 + 0.04431X, and the detection limit was 1.0 × 10^−10^ mol/L. The quantitative analysis of Hg^2+^ concentration in samples based on this linear relationship indicated that this probe had a good application prospect in detecting Hg^2+^ in water samples.

### 2.8. Detection of Water Samples

Based on the superior electivity and sensitivity of the RFN in the buffer solutions, we further applied it to the detection of actual water samples. The water samples in this experiment were taken from Tiannan Lake and the tap water. Before the experiment, the water sample was centrifuged and its supernatant was taken for the experiment. A 500 μL of Tiannan Lake water sample or tap water sample were mixed with 500 μL of a certain concentration of mercury ion solution, and then mixed with a 150 μL of the RFN to make the total volume of 4 mL. As shown in Table 1, the standard recovery rate of mercury ions were detected at room temperature, and the recovery was calculated to be between 98.15% and 100.45% by linear regression equation, indicating that the method had good accuracy.

## 3. Materials and Methods

### 3.1. Reagents and Materials

All the starting materials were used without further purification. AgNO_3_, HAuCl_4_, NH_3_·H_2_O were obtained from Sinopharm Chemical Regent Co. Ltd. (Shanghai, China). Tetraethyl orthosilicate (TEOS), 1-[3-(dimethylamino) propyl]-3-ethylcarbodiimide (EDC), 3-aminopropyl-trimethylsilane (APTES), and fluorescein isothiocyanate (FITC) were purchased from Aladdin Chemicals Co. Ltd (Shanghai, China). For all aqueous solutions, high-purity ultrapure water from a Millipore (18.2 MΩ·cm) system was used throughout the experiments.

### 3.2. Measurements

FRN were characterized by using high-resolution transmission electron microscopy (Hitachi, Japan). Ultraviolet–visible (UV–Vis) absorption spectra were acquired on a Cary 5000 spectrophotometer (Agilent, Palo Alto, CA, USA) coupled with a 1.00 cm quartz cell. All Fluorescence spectra were performed under λ_ex_ = 380 nm with FluoroMax-4 (Horiba, Japan).

### 3.3. Synthesis of FITC–SiO_2_ Microspheres

The FITC–SiO_2_ microspheres was prepared according to a modified method that was previously reported [20], 1 mg of FITC was weighed then dissolved in 5 mL of 1-hexanol, and 100 μL of APTES was added in a small bottle. After that, the resulting mixture was stirred at medium speed for 12 h in the dark to obtain the FITC–APTES conjugate. Following this, 7.5 mL of cyclohexane, 1.4 mL of 1-hexanol, and 1.77 mL of TX-100 were added to a small bottle sequentially under low-speed stirring and stirred for 15 min; 60 μL of concentrated ammonia was added and stirred at medium speed for 15 min; 400 μL of FITC–APTES conjugate and 92 μL of TEOS were added and stirred for 12 h in the dark; the product was centrifuged at 8000 rpm for 8 min and washed twice with absolute ethanol to obtain FITC–SiO_2_ microspheres.

### 3.4. Synthesis of AuNCs

AuNCs were prepared following previous method and minor modified [18]. 10 mL of 10 mmol/L aqueous HAuCl_4_ solution was added to 10 mL of 50 mg/mL BSA solution under vigorous stirring; 1 mL of NaOH solution (1 mol/L) was then introduced to adjust pH, and the mixture was incubated at 37 °C for 12 H. The solution was then dialyzed in double distilled water for 48 H to remove the unreacted HAuCl_4_ and NaOH. The final solution was stored at 4 °C for further use.

### 3.5. Synthesis of Amino-Modified FITC–SiO_2_ Microspheres

1 mL of the above synthetic FITC–SiO_2_, 6 mL of ethanol, 100 μL of concentrated ammonia, and 15 μL of TEOS and APTES were added to a small glass bottle, stirred for 2 h, and washed twice with high purity water (8000 rpm, 8 min) to obtain amino-modified FITC–SiO_2_ microspheres.

### 3.6. Synthesis of RFN

**Scheme 1:**
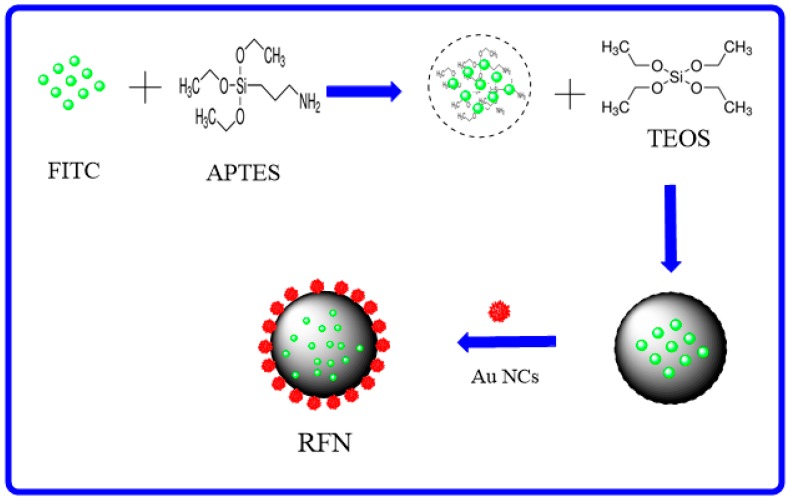
Schematic illustration for preparation of the RFN.

Consequently, 20 mg of EDC was weighed and dissolved in 1 mL of high-purity water, 4 mL of AuNCs was added, and the pH value of the reaction system was adjusted to 5.7 with 0.01 mol/L HCl. After stirring for 30 min, 1 mL of NH_2_–FITC–SiO_2_ was added to the system, the pH value of which was adjusted to 9.0 with 0.01 mol/L NaOH. The reaction system was stirred for 12 h in the dark, then centrifuged twice with high-purity water to remove unreacted materials and collected with 1 mL of high-purity water to obtain RFN, as shown in Scheme 1.

## 4. Conclusions

In this study, the RFN was successfully synthesized and applied to the detection of mercury ions in water samples. The RFN had a simple synthesis procedure, high sensitivity, good stability, and strong anti-interference. The fluorescence intensity ratio of the RFN achieved the best effect at a pH value of 9.15. The RFN could detect Hg^2+^ in water simply and quickly, and the detection concentration ranged from 1.0 × 10^−10^ mol/L to 1.0 × 10^−8^ mol/L, with the detection limit of 1.0 × 10^−10^ mol/L. The advantages of good stability and high selectivity make the RFN suitable for monitoring heavy metal in waters. This method has a good application prospect for environmental protection.

## Figures and Tables

**Table 1:** The RFN for the detection of Hg^2+^ in Tiannan lake water and tap water.

Sample	Hg^2+^ Concentration (10^−10^ mol/L)			
	Present Method	Added	Total	Recovery
Tiannan lake	4.88	5	9.84	99.2%
		20	24.51	98.15%
Tap water	1.49	5	6.46	99.4%
		20	21.58	100.45%

## Supplementary Materials

The Supplementary Materials are available online.
