# Supplementary material for: Ratiometric Fluorescent Nanoprobe for Highly Sensitive Determination of Mercury Ions
Source: Molecules. 2019 Jun 19;24(12):2278. doi: 10.3390/molecules24122278 (PMC6631757; doi:10.3390/molecules24122278)
Supplement: Supplementary file 1 [file molecules-24-02278-s001.pdf]

supporting information

Preparation of ratiometric fluorescent nanoprobe and its application in the determination of mercury ions

Zhihui Luo[1,2,4], Hui Xu[1,4], Baogui Ning[2], ZeBin Guo[1,4], Na Li[2], Lina Chen[2], Guobao Huang[2], Charlie Li[3] and Baodong Zheng[1,4]

1 College of Food Science, Fujian Agriculture and Forestry University, Fuzhou, Fujian 350002, China
2 Guangxi Key Laboratory of Agricultural Resources Chemistry and Biotechnology, Colleges and Universities Key Laboratory for Efficient Use of Agricultural Resources in the Southeast of Guangxi, College of Chemistry and Food Science, Yulin Normal University, Yulin, Guangxi 537000, PR China.
3 Department of Environmental Toxicology, University of California-Davis, Davis, CA 95616, USA
4 Engineering Research Center of Marine Living Resources Integrated Processing and Safety Risk Assessment, Fuzhou, Fujian 350002, P. R. China
* Correspondence: zbdfst@163.com (B. Zheng); csjli@ucdavis.edu (C. Li)

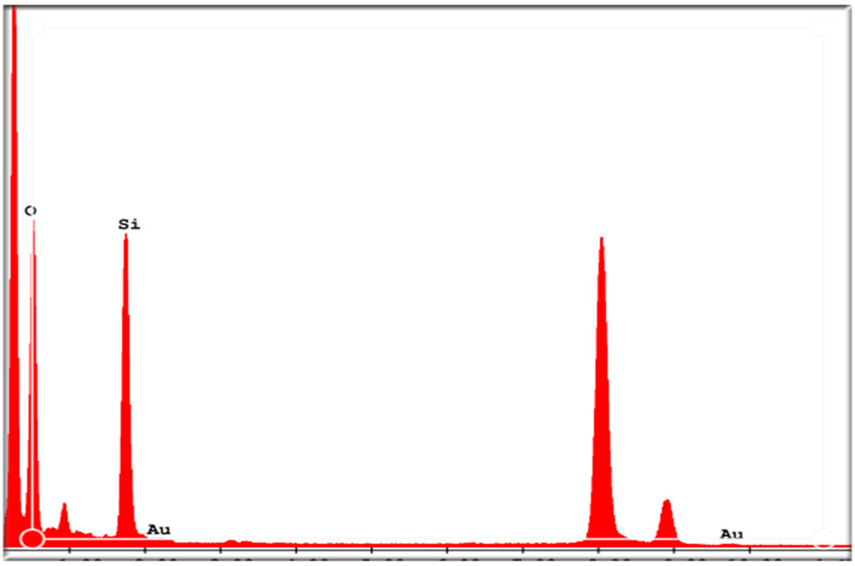

Figure S1. Electron scattering spectroscopy photo of ratiometric fluorescent nanoparticles.
